# 1282. Evaluation of Opportunities for Implementing OVIVA Criteria on Patients with Bone and Joint Infections (BJIs) in Outpatient Parenteral Antimicrobial Therapy (OPAT)

**DOI:** 10.1093/ofid/ofad500.1121

**Published:** 2023-11-27

**Authors:** Garrett Crawford, Michelle Wasan, Jessa Brenon, Caitlin Soto, William F Wright, Sara C Keller

**Affiliations:** The Johns Hopkins Hospital, Baltimore, Maryland; The Johns Hopkins Hospital, Baltimore, Maryland; Johns Hopkins Home Care Group, Baltimore, Maryland; The Johns Hopkins Hosiptal, Baltimore, Maryland; Johns Hopkins University, Baltimore, Maryland; Johns Hopkins University School of Medicine, Baltimore, MD

## Abstract

**Background:**

Historically, long term intravenous (IV) antimicrobial therapy has been considered superior to oral (PO) for the management of BJIs. In recent years, this concept has been challenged with studies showing similar efficacy and safety between PO and IV therapy. The purpose of this study is to evaluate areas for improvement within Johns Hopkins Medicine (JHM) in utilizing oral antibiotics for the treatment of BJIs as outlined in the OVIVA trial.

**Methods:**

A multicenter, retrospective study was performed of adult patients with BJIs discharged from The Johns Hopkins Hospital and Johns Hopkins Bayview Medical Center followed by the JHM OPAT Service between February 1, 2021 and February 1, 2022. The following was collected on all patients: demographics, comorbidities, site of infection, OPAT-related adverse events (AEs), 30-day readmissions, emergency department (ED) visits, source control, microbiological data, antibiotic regimen, and treatment outcomes. The primary outcome is the proportion of patients treated for BJIs with OPAT who would have qualified for oral antibiotics according to OVIVA trial criteria. Secondary outcomes include the rate of OPAT-related AEs and treatment outcomes (30-day readmissions and ED visits, 6-month treatment failure, early IV to PO transitions, and durations of therapy). Descriptive statistics were used to summarize demographics and the primary and secondary outcomes.

**Results:**

Among the 350 patients with BJIs, 221 patients met inclusion criteria. For the primary outcome, 169 (76.4%) met oral eligibility criteria with sulfamethoxazole-trimethoprim, tetracyclines, and cephalosporins being the most common PO options. There were 35 total OPAT-related AEs (24 [14.2%] in the PO eligible group and 11 [21.2%] in the PO ineligible group). There were 15 readmissions (11 [6.5%] in the PO eligible group and 4 [7.7%] in the PO ineligible group) and 17 ED visits (13 [7.7%] in the PO eligible group and 4 [7.7%] in the PO ineligible group). For 6-month treatment failure, there were 29 total occurrences (23 [13.6%] in the PO eligible group and 6 [11.5%] in the PO ineligible group).

**Conclusion:**

Based on OVIVA trial criteria, the majority of evaluated patients could have utilized oral antibiotics, which could have resulted in fewer OPAT-related AEs, readmissions, and ED visits.

**Disclosures:**

**Sara C. Keller, MD, MPH, MSPH**, Pfizer: Advisor/Consultant

